# The distribution of submerged macrophytes in response to intense solar radiation and salinity reveals hydrogen peroxide as an abiotic stress indicator

**DOI:** 10.1038/s41598-023-30487-1

**Published:** 2023-03-20

**Authors:** Takashi Asaeda, Md Harun Rashid, Xia Liping, Lekkala Vamsi-Krishna, Abner Barnuevo, Chihiro Takeuchi, Mizanur Rahman

**Affiliations:** 1 Saitama University, Saitama, Japan; 2 Department of Agronomy, Bangladesh Agricultural University, Mymensingh, Bangladesh; 3 Diamond Company, New Delhi, India

**Keywords:** Environmental chemistry, Environmental impact

## Abstract

The feasible condition for submerged macrophyte growth is hard to understand as many environmental factors contribute to establishing macrophyte distribution with different intensities generating excess reactive oxygen species (ROS). Among various kinds of ROS, hydrogen peroxide (H_2_O_2_) is relatively stable and can be measured accurately. Thus, for the quantification of submerged macrophyte species, H_2_O_2_ can be used to evaluate their distribution in a lake. Submerged macrophytes, such as *Potamogeton anguillanus*, were abundant in Lake Shinji. The largest biomass distribution was around 1.35 m deep, under low solar radiation intensity, and nearly no biomass was found less than 0.3 m deep, where solar radiation was high. Tissue H_2_O_2_ concentrations varied in response to the diurnal photosynthetically active radiation (PAR) intensity, which was followed by antioxidant activities, though slightly delayed. Laboratory experiments were conducted with different PAR intensities or salinity concentrations. A stable level of H_2_O_2_ was maintained up to about 200 μmol m^−2^ s^−1^ of PAR for 30 days, followed by a gradual increase as PAR increased. The H_2_O_2_ concentration increased with higher salinity. A change in Chlorophyll *a* (Chl-*a*) concentration is associated with an altering H_2_O_2_ concentration, following a unique negative relationship with H_2_O_2_ concentration. If H_2_O_2_ exceeded 45 μmol/gFW, the homeostasis collapsed, and H_2_O_2_ and Chl-*a* significantly declined afterward. The above findings indicate that H_2_O_2_ has a negative effect on the physiological condition of the plant. The increase in H_2_O_2_ concentration was prevented by antioxidant activities, which elevated with increasing H_2_O_2_ concentration.

## Introduction

Macrophytes play important ecological roles, and they can colonize a variety of aquatic environments^1^. Their colonization in different environments is governed by an array of external factors and the abilities of a particular macrophyte to cope with that environment^2–5^. It has always been a key challenge for aquatic ecologists to discover the environmental factors that drive the richness and distribution of these plants. Among these external factors, underwater light intensity is very important. It affects macrophyte growth and biomass production^6^, reproduction^7^, distribution^3^, and phenotypic variation^8^. Underwater light intensity at a water stratum is governed by depth^9^, canopy openness^3^, or water turbidity^10^.

In freshwater ecosystems, roughly 10% of the global radiation is reflected at the air–water interface, and a significant portion is attenuated with depth, resulting in low light conditions in most deep aquatic habitats^11^. Thus, water depth and low light intensity inhibits the colonization of the plants^12–15^. High solar radiation damages the photosynthetic apparatus at the same time and degrades the photosynthesis rate, called photoinhibition^16,17^. Thus, the too-shallow water seems to be unsuitable for the colonization of submerged plants, though it is not much discussed.

Many studies have already investigated submerged plants to characterize their photoinhibition by fluorescence yield^8–20^. This technique indicates the energy flow rate utilized in the photosynthesis process as a result of the radiation’s effect on the thylakoid’s photosynthesis apparatus. However, it is difficult to quantify the process compared with other abiotic stresses. Solar radiation’s excessive energy stresses the plant cell organelle and generates the ROS in the chloroplast^21^. Various physiological and environmental factors directly affect the photoproduction of ROS. The photoproduction rate increases and is directly proportionate to photon intensity, which is required for CO_2_ assimilation^21^. Singlet oxygen, generated at PSII, damages the PSI and PSII apparatuses^22,23^. In the electron transport chain, the superoxide radical (O_2_^**.**−^) is generated due to the partial reduction of O_2_ or as a result of a high energy supply. The excessive number of electrons generates the ROS super oxide anion (^.^O_2_^−^) from oxygen. They are dismutated to H_2_O_2_ by the activity of super oxide dismutase (SOD). Singlet oxygen and H_2_O_2_ inhibit the production of the D1-protein, which otherwise reactivates the damaged PSII^24^. Other stress types, such as unsuitable temperatures and anoxic conditions, also generate ROS on other organelles^25^, which then disrupt the photosynthesis, affecting colonization or growth^25–27^.

Salinity is an important abiotic factor limiting the development and primary production of plants^28^, since it may cause ionic disequilibrium and oxidative stress^29^. It also determines the distribution and colonization of aquatic macrophytes. Oxidative stress is an important indicator of salt stress, since the plant under the effect of salt may increase the production of ROS and suffer cell damage^30,31^. H_2_O_2_ is one of the main cellular metabolites that, at low concentrations, acts as an important signaling factor in the cell defense metabolism^32^. On the other hand, H_2_O_2_ at high concentrations and in the presence of transition metals may generate the hydroxyl radical (OH⋅) that can transpose and disintegrate cell membranes^33^. Therefore, the concentration of H_2_O_2_ is highly likely to become a useful indicator of oxidative stress to evaluate colonization ability^34,35^.

Under stress conditions, plants deal with the effects of ROS through an enzymatic complex^36,37^, triggering a non-enzymatic antioxidant mechanism^38,39^. Antioxidant activities depend on the ROS concentration, while in the recovery process, the antioxidant, particularly catalase activities, is delayed compared to the ROS concentration^40^.

Both laboratory and field experiments were conducted to elucidate the relationships between photoinhibition and light intensity^41–43^. Unlike the regulated condition of the laboratory experiments, the solar radiation intensity in the field condition varies from time to time. Usually, it is higher than that of laboratory experiments^41^. Plants should be more susceptible to the combined effect of intensive solar radiation and salinity. Thus, field observation is crucial to understand the daily effect of solar radiation on the photosynthesis and growth rate of submerged plants. We hypothesize that under intense solar radiation and salinity conditions, the homeostasis between tissue H_2_O_2_ generation and antioxidative mechanisms is disrupted, limiting the growth and biomass of submerged macrophytes^44^. In this study, H_2_O_2_ concentration and the intensity of antioxidant activities were measured under high solar radiation on a summer day, particularly focusing on photoinhibition to introduce the biomass distribution pattern along the shoreline of a lake. Also, the biomass distribution of a submerged macrophyte species, *P. anguillanus*, was observed in a brackish lake. Its trend was analyzed from the viewpoint of the tissue H_2_O_2_ concentration and antioxidant activities with the aid of laboratory experiments.

## Results

### Field observation

As a result of excessive turbulence caused by river inflows and winds, a clear halocline formed at about 4.0 m deep from the surface^45^. The surface layer salinity was 2.5‰, and the lower-layer salinity was measured as about 7‰, similar to other reports^46,47^. Other parameter measurements, such as surface water temperature (26.6 °C), pH (7.4), conductivity (8.71 μS/m), and DO (9.1 g/L), were also obtained.

### *P. anguillanus* biomass and PAR distribution along water depth

**Figure 1:**
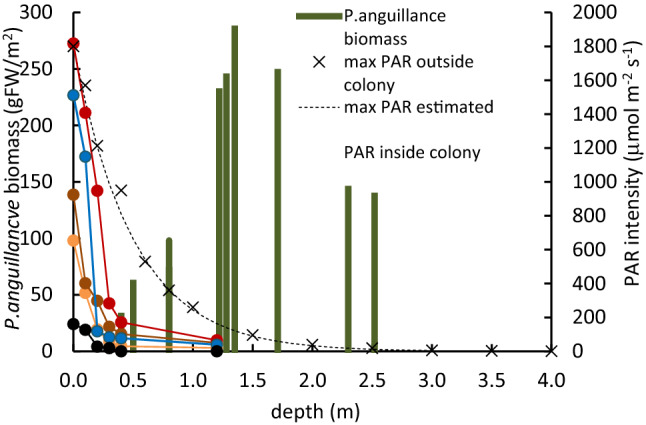
Diurnal distribution of PAR and biomass of *P. anguillanus* at different depths in Lake Shinji.

There was no gradient in oxygen and other elements up to about 4 m depth (data not presented), except for PAR intensity. The PAR intensity attenuated exponentially, with 2.0 (1/m) of the constant attenuation coefficient, which was similar to the other report values. In the *P. anguillanus* colonized zone, the bottom to about 0.3 m depth was occupied by *P. anguillanus* tissues. Figure 1 shows a rapid decline in PAR intensity below the *P. anguillanu*s colony at 0.25 to 0.4 m deep. Horizontally, no biomass of *P. anguillanus* was observed from the shoreline to 0.3 m depth. With increasing depth, the biomass increased up to 1.35 m depth, then gradually decreased at deeper sites (Fig. 1). No biomass was observed at 3.5 m depth. The maximum shoot length was slightly longer than 3 m. Outside the colony, the maximum bottom PAR intensity at 0.3 m depth was about 1000 μmol m^−2^ s^−1^ and was about 150 μmol m^−2^ s^−1^ at 1.35 m depth. The H_2_O_2_ contents, the Chl-*a* concentration and the total antioxidant activity of *P. anguillanus* between these two sites, were highly correlated with each other (*r* = 0.877, *p* < 10^−5^ for the H_2_O_2_, *r* = 0.48, *p* < 0.01, for the Chl-*a*, and *r* = 0.68, *p* < 0.01 for the antioxidant activity).

**Figure 2:**
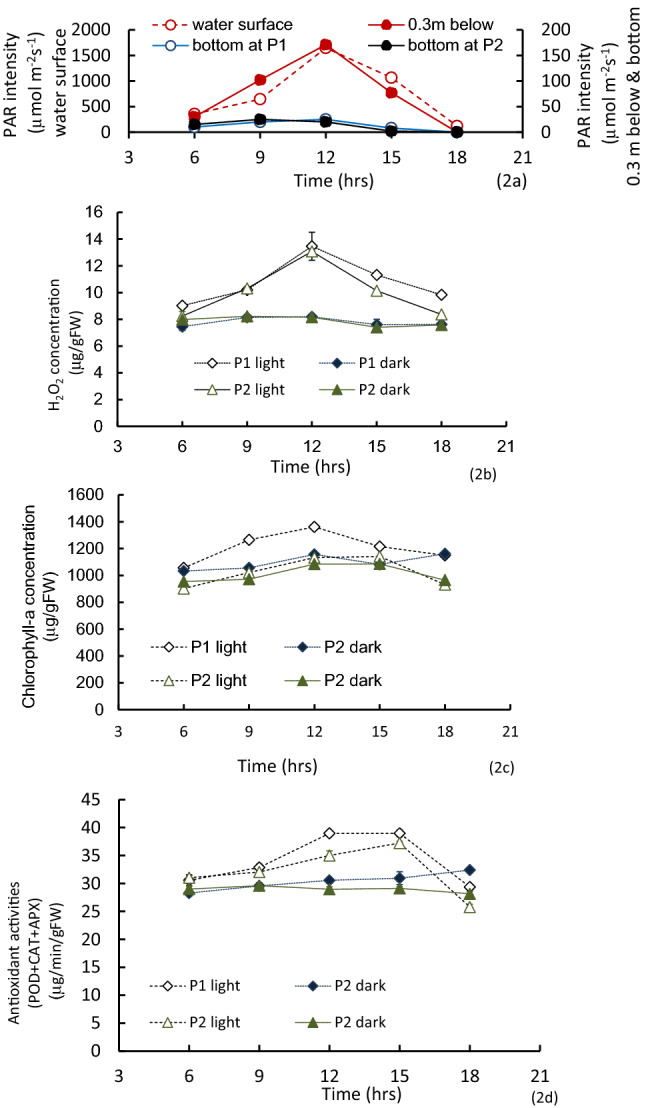
The variations of PAR intensity at water surface, 0.3 m below the surface, at bottom of sampling points, P1 and P2, H_2_O_2_ concentration of tissue samples at light- exposed and shaded samples of P1 and P2 points, and the sum of three antioxidant enzyme activities of tissue samples at light-exposed and shaded samples of P1 and P2 points. Vertical bars indicate the standard deviation.

In the light exposed condition, leaf tissue H_2_O_2_ contents at the top of the colony were approximately 8 μmol/gFW in the early morning. This measurement increased until 12 a.m. (13.07 μmol/gFW) and then gradually decreased to the early morning value, following the variation of PAR intensity (Fig. 2a). While, Chl-*a* concentration was slightly less than 900 mg/gFW in the early morning, H_2_O_2_ peaked approximately 1100 μmol/gFW, at 12:00 noon, then declined as the time progresses. The total antioxidant activity, the sum of POD, CAT and APX, was about 30 μmol/min/gFW in the early morning, after that, rose until 40 μmol/min/gFW at 3:00 pm, then decreased (Fig. 2c,d).

In contrast, these values in shaded *P. anguillanus* tissues did not indicate any diurnal variation (*p* = 0.151 for H_2_O_2_; *p* = 0.576 for Chl-*a*; *p* = 0.968 for the total antioxidant activity). The values in both sites were nearly constant throughout the day (Fig. 2). Different light intensities and H_2_O_2_ concentrations were significant in the Kruskal–Wallis test (*p* < 0.01).

### The effect of high PAR and salinity exposure

**Figure 3:**
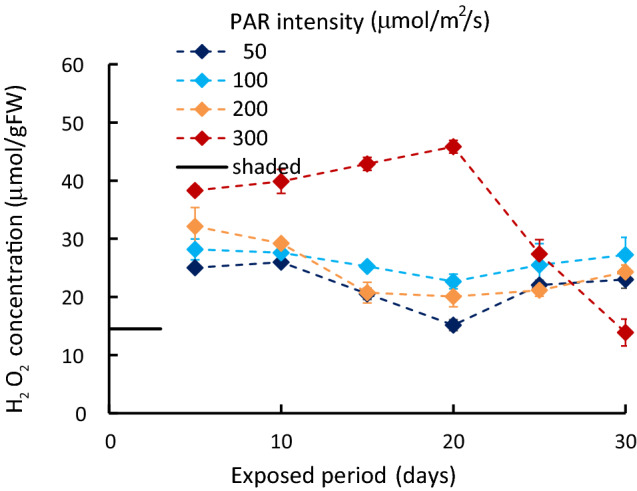
The variation of H_2_O_2_ concentration at 1:00 p.m. exposed to different PAR intensities. Vertical bars indicate the standard deviation.

In the laboratory experiments, the initial value of tissue H_2_O_2_ concentration was approximately 13 μmol/gFW. The concentration was exposed to different light intensities beginning at 6:00 a.m. By 1:00 p.m., the H_2_O_2_ concentration had significantly increased by about 15–25 μmol/gFW (*p* < 0.001). With 50–200 μmol m^−2^ s^−1^ of PAR intensity, the value at 1:00 p.m. remained nearly constant throughout the experimental period, although it slightly declined around day 20 and recovered afterward. However, with 300 μmol m^−2^ s^−1^ of PAR intensity, the value at 1:00 p.m. had increased to about 45 μmol/gFW, then rapidly declined (Fig. 3).

**Figure 4:**
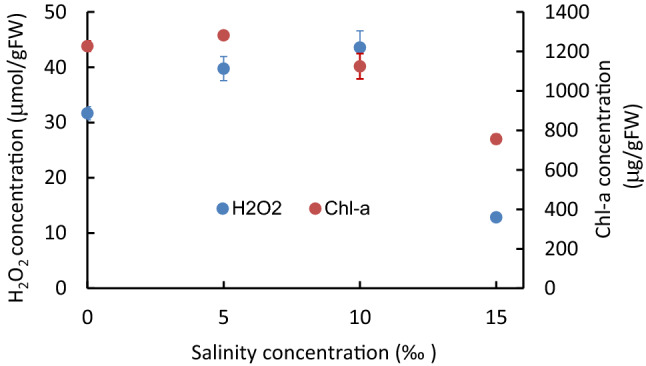
The variation of H_2_O_2_ and Chl-*a* concentrations after 7 days’ exposure to the different salinity concentrations. Vertical bars indicate the standard deviation.

Concerning salinity exposure, tissue H_2_O_2_ concentration increased in direct proportion to salinity concentration until 10‰, where the value was 45 μmol/gFW (*p* < 0.05), then significantly (*p* < 0.01) declined with further higher salinity. On the other hand, Chl-*a* concentration declined from 5‰ onwards with an increasing salinity level (Fig. 4) (*p* < 0.05, in the Kruskal–Wallis test).

**Figure 5:**
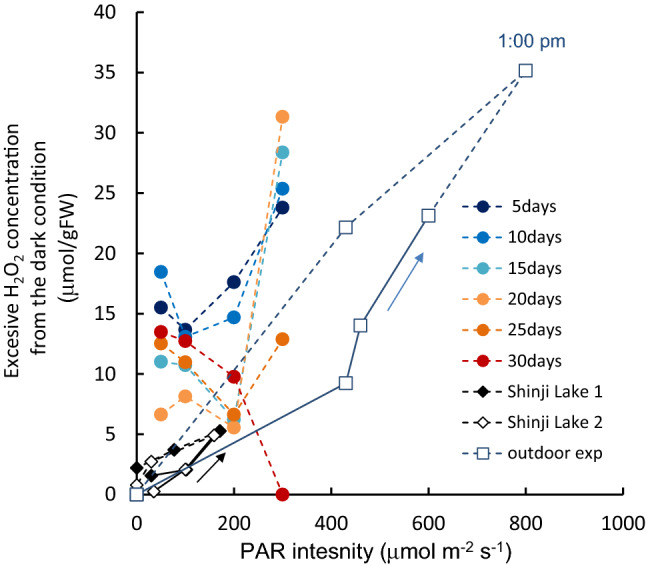
The H_2_O_2_ increment due to the PAR intensity from the values of samples in dark condition. In Shinji Lake, and outdoor exp., straight line: morning (PAR increasing), and dashed line: afternoon (PAR decreasing).

**Figure 6:**
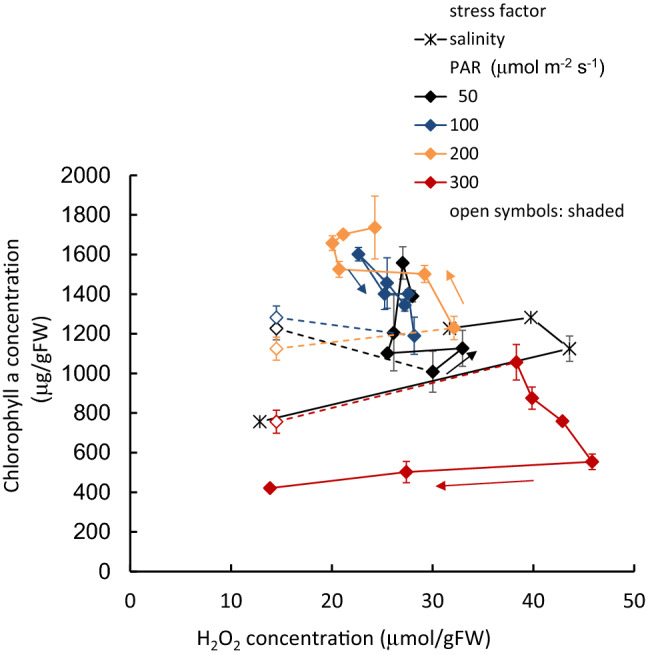
Chl-*a* concentration of tissues with respect to the H_2_O_2_ concentration. Dashed lines indicate the starting dark condition to the exposure of each PAR intensity, and straight lines indicate the variation due to the experimental period.

The increase in the H_2_O_2_ concentration of the light-exposed samples from shaded sample values is shown as a function of the PAR intensity in Fig. 5, together with field and outdoor experiment results. The H_2_O_2_ concentration was generally higher under simultaneous PAR intensity. It was slightly lower with samples in the increasing PAR stage than those in the decreasing stage. The 1:00 p.m. value generally increased with increasing PAR intensity. After 25 days, however, it significantly decreased from 100 μmol m^−2^ s^−1^ of PAR onwards (*p* < 0.001). The Chl-*a* concentration changed as the H_2_O_2_ concentration increased or decreased, keeping a unique relationship between H_2_O_2_ and Chl-*a* concentrations in either PAR or salinity altered (Fig. 6).

**Figure 7:**
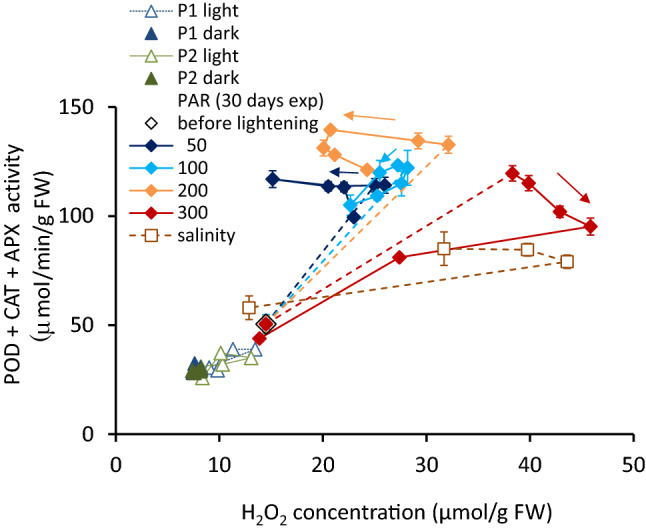
The sum of three antioxidant activities as a function of H_2_O_2_ concentration. Vertical bars indicate the standard deviation. Connecting dashed line indicates the starting dark condition to the exposure of each PAR intensity; straight lines indicate the variation due to the experimental period.

The antioxidant activity, as the sum of three enzyme activities, is shown in μmol/min/gFW (Fig. 7). Antioxidant activities were generally correlated positively with the instantaneous H_2_O_2_ concentration (*r* = 0.671, *p* < 0.01). Diurnally, the antioxidant activity of the light-exposed samples were high during midday. The total activity was higher in the early afternoon, and the peak values were delayed compared to the PAR and H_2_O_2_ variations. On the other hand, shaded samples kept nearly the same level at 6:00 a.m. throughout the day (Fig. 2c).

## Discussion

### Biomass distribution in Lake Shinji

In the previous report, *P. anguillanus* was observed from June to December and was found up to 2.5–3.0 m depth, with the highest biomass at 1.5 m depth^48^. In the present study, there was no biomass up to 0.3 m depth, and the biomass was highest, 300 gDW/m^2^, at 1.35 m depth, then decreased in the deeper zones. Biomass distribution follows a perfect agreement. The salinity level of the lake water fluctuated depending on the inflow volume and was higher with low inflow and lower with high inflow^46,47^. The salinity at 0.5 m above the bottom of the lake center (6 m depth) was about 3.6‰ (1.0–7.4‰) at the bottom in average for the last 20 years, and the fluctuation was relatively small at 0.6–0.7‰^45^. It may affect the distribution of *P. anguillanus* in Lake Shinji^47^. However, compared with the present results on the tolerance of this species against the salinity concentration at *P. anguillanus* colonized depth, this salinity level does not contribute much to reducing biomass, even in the deep zone (Fig. 4). Most of turbidity flows in during flood periods, from the Hii River, located at the western end of the lake and does not affect the central part of the lake. Any specific sightings such as herbivores, etc., are recognized in these days^49^.

The major stress seems to be high solar radiation combined with salinity stress^50^. High mechanical disturbance inhibits the colonization of submerged plants near the shoreline^51–53^. However, a large number of emergent plants were naturally colonized along the shoreline decades ago^54,55^, and *Phragmites australis* colonies have now been widely recovered in some areas. Mechanical disturbance is not a major reason to inhibit *P. anguillanus* in the shallow zones.

The generation rate of superoxide radicals (^**.**^O_2_^−^) depends on the uptake rate of inorganic carbon. Exposure to 2 μmol of dissolved inorganic carbon is sufficient for photosynthesis under even 1200 μmol m^−2^ s^−1^ of radiation^56^. The relatively high inorganic carbon was dissolved in the lake water due to the wave activities by the strong winds and with around pH 7, where a sufficient amount of CO_2_ existed (pCO_2_ ≡ 150–1000, DIC ≡ 600–700). Thus, the excessive H_2_O_2_ was considered due to the high solar radiation effect rather than the lack of carbon dioxide.

At 0.3 m depth, the light intensity was 600 μmol m^−2^ s^−1^, and at 1.35 m depth, 30 μmol m^−2^ s^−1^. As a result of excessive solar radiation, *P. anguillanus* cannot extend to near water surface in deep sites, and grow after germination in shallow sites, which may explain the low biomass at less than 0.3 m depth. However, the germination of *P. anguillanus* is capable at very low light or even dark conditions, then can extend to 30–50 cm long even in dark conditions^57^. Thus, 2.5–3.0 m is the maximum depth in which the plant canopy can reach until sufficient photosynthesis is expected after germination and continue to grow, although PAR at the bottom is too low. However, the highest biomass was achieved at 1.35 m depth where the preferable light intensity could be received at the bottom just after germination.

### The response of hydrogen peroxide to solar radiation

Under environmental stresses, the ROS generated in the various organelles, chloroplast, mitochondria, and peroxisome damages proteins, genes, and lipids, although antioxidant activities scavenge them^58^. H_2_O_2_ is a major ROS, either directly generated or generated in many organelles from superoxide radicals by SOD activity. Experimental studies by many laboratories have been conducted to determine the relationship between the H_2_O_2_ concentration and the various types of stress intensities^59–61^.

Unlike laboratory experiments, which are controlled intentionally, there are various environmental stressors in the real field, and their intensities vary randomly or regularly with different time scales. This study shows that the H_2_O_2_ concentration increases with elevating stress intensity, then affects Chl-*a* concentration, following a nearly unique relationship irrespective of stress types and their processes. However, there is a slight difference between the increasing and decreasing stress intensity processes. The response is sufficiently rapid compared with the change in the environment. At the same time, if the H_2_O_2_ concentration exceeded 45 μmol/gFW, it significantly declined afterward, even against the higher stresses. The high H_2_O_2_ concentration deteriorates the physiological condition without generating further H_2_O_2_^44,62^.

Low H_2_O_2_ concentration is generated with sufficiently low PAR, and the Chl-*a* concentration fluctuates positively with the PAR (data are not shown). It may be due to an adaptation to increase productivity. In contrast, high Chl-*a* concentration increases the photosynthesis rate, generating more H_2_O_2_ and deteriorating the photosynthesis apparatus. Thus, increased H_2_O_2_ concentration negatively alters the Chl-*a* concentration in highly stressed conditions^44^. The H_2_O_2_ concentration is likely to indicate the environmental stress intensity on plants and productivity^63^. This study showed the significant negative effect of H_2_O_2_ on *P*. *anguillanus*. Other submerged macrophytes, such as *Egeria densa*^35,63^, *Elodea nuttallii*^63^, *Potamogeton crispus*^63^*, Ceratophyllum demersum*^40^*, *and *Vallisneria asiatica*^60^*, *had similar findings.

### The effect of antioxidant activities

The tissue H_2_O_2_ concentration at a particular time is determined as the balance between the generation rate and the scavenging by antioxidant activities. This concentration works as a signal to activate antioxidant behavior. After receiving H_2_O_2_ signals, antioxidants are formed and activated to scavenge the effect of H_2_O_2_^25,64,65^. Varying light intensity delays the behaviors of the antioxidant activities and results in H_2_O_2_ variations. The present study indicated that the delay period was 2–3 h compared to the variational pattern of the H_2_O_2_ concentration, although this period depended on the analysis frequency. Then, the H_2_O_2_ concentration becomes higher in the early afternoon, not exactly when the solar radiation is highest (Fig. 2).

### Comparison with Chl-*a* fluorescence behavior

PSII photochemical efficiency is given by Chl-*a* fluorescence (*F*_*v*_/*F*_*m*_). Therefore, the photoinhibition effect was studied using an indicator of photosynthetic quantum yield. The relationship between the H_2_O_2_ concentration and the *F*_*v*_/*F*_*m*_ ratio is unclear. The linear relation of the photosynthetic rate with the *F*_*v*_/*F*_*m*_ ratio is given^18^, and no reduction of *F*_*v*_/*F*_*m*_ was recorded to 70 μmol m^−2^ s^−1^ of PAR^66^ for *Egeria densa, E. nuttallii*, and *Myriophyllum heterophyllum*. A quick reduction was observed in 3 h after exposure to 100 μmol m^−2^ s^−1^ light. The fraction of PAR is approximately 0.45 of the total solar radiation. Thus, 70 μmol m^−2^ s^−1^ PAR corresponds to about 160 μmol m^−2^ s^−1^ total solar radiation, corresponding to the solar radiation of the lowest H_2_O_2_ concentration. The quick reduction of fluorescence has been reported^18^, such that the fluorescence reduced quickly (about 20 min) after exposure to the high light, 500–1000 μmol m^−2^ s^−1^. There was a relatively slow (about 6 h^19^) yield recovery in the shade after 30 min solar radiation exposure. Though the period is slightly different, the response time trend agrees with the variation tendency of the H_2_O_2_ concentration.

## Conclusions

Tissue H_2_O_2_ concentration becomes higher in unsuitable conditions where oxidative stress is high. Excessive light intensity and elevated salinity increase H_2_O_2_ concentration. Chl-*a* concentration has a unique negative correlation with H_2_O_2_ concentration. If H_2_O_2_ exceeds a threshold value, physiological homeostasis collapses, and H_2_O_2_ and Chl-*a* concentrations significantly decline. Therefore, the largest biomass in a lake is distributed in a slightly deep zone, where sufficient solar radiation is received after an extended period, rather than in a shallow zone, where solar radiation is high at the bottom.

## Methodology

### Ethical permission

This study has been approved by the Ministry of Land, Infrastructure, Transport, and Tourism of Japan (the River Works Technology Research and Development program "The research on the development of sustainable ecosystem harmonized with human activity", 2016–2020, Project leader: Prof. Masumi Yamamuro) and by Shimane Prefecture "Hozen-Saisei Kyogikai of Lake Shinji", 2016-. The collection of plant samples also followed international guidelines and legislation and approved by the above authority.

### Field sampling

Lake Shinji is 79.25 km^2^ wide and 4.5 m deep on average. Located in western Japan (35°27′N, 132°57′E), it is a brackish water lake with 4–8‰ salinity at the bottom. The shoreline is composed of sandy sediment. TN and TP concentrations have been relatively stable during the last three decades, with TN of 0.82–0.13 mg L^−1^ and TP of 0.01–0.23 mg L^−1^, respectively^44^. Until around 2010, Lake Shinji had no significant submerged vegetation growth. Since then, however, macrophytes (e.g., *P. anguillanus* and *P. panormitanus*) have grown, and presently they thickly cover nearly half of Lake Shinji’s shoreline.

Macrophyte distribution was observed along the lake’s entire shoreline to confirm that the biomass distribution was highly dependent on depth. Then, two typical sites were selected at the northern shoreline, where a pure colony of *P. anguillanus* was formed. Detailed sampling was conducted on August 22–24, 2017. At each site, a random transect was taken perpendicularly to the shoreline toward the deep zone, and *P. anguillanus* samples were taken from 50 cm × 50 cm quadrats placed every 20 m along the transect. All plants in a transect were sampled to obtain the biomass distribution with respect to the depth. The samples were sealed in plastic bags to transport to the laboratory. Water quality parameters at the sampling sites were measured by a water quality meter (U-51, Horiba, Kyoto, Japan). In the laboratory, the collected plant samples were oven-dried until no weight change was registered. Then, the dry weight was measured.

The in situ experiments were conducted at two sites with typical colonies of *P. anguillanus*, where *P. anguillanus* was exposed to either (a) natural solar radiation, or (b) dark adapted. The dark exposure treatment was performed by placing a black plastic sheet (3 m × 3 m) floating over the *P. anguillanus* colonies for 30 min^35,67^. The 30 min pre-dark period was determined from laboratory experiments, which were conducted to determine the optimum pre-darkness duration. The plastic sheets were tied to fixed metal poles inserted in the bed, allowing the sheets to float on the water surface without causing mechanical disturbances to the macrophytes or altering the water flow. Chemical analysis samples were taken every 3 h from 6:00 a.m. until 6:00 p.m. at two pure *P. anguillanus* colonies 1.22 m deep (P1, 35°28′25.93″N, 132°57′2.14″E) and 1.35 m deep (P2, 35°28′25.13″N, 132°57′2.09″E). PAR intensity was measured by a portable quantum flux meter (Apogee, MQ-200, United States) above and below the water surface and at every 10 cm deep up from the bottom. Then, healthy plants were sampled at the top of the canopy, approximately 30 cm deep from the water’s surface, both from the colonies exposed to solar radiation and darkness treatment. The samples were tightly sealed in a plastic bag and stocked either in a cooling box with dry ice (about − 70 °C) or air temperature to transport to the laboratory. Chemical analyses were conducted first.

### Laboratory experiments

#### Culture procedure of *P. anguillanus*

A healthy stock of *P. anguillanus* was collected from Shinji Lake. The collected plants were cleaned with distilled water to remove debris, and we utilized tweezers to carefully separate the attached algae. Then, the plants were cultured in a glass tank under laboratory conditions (25 ± 2 °C, 12/12 h photoperiod, PAR intensity 100–150 μmol m^−2^ s^−1^) for approximately three months. In all experiments, the light was provided by LED straight lights (Model LT-NLD85L-HN, OHM Electric INC, Japan). The tanks used for experiments were wrapped with a reflective sheet so that every part of plant tissue was homogeneously exposed to the same light intensity. Commercial sand (D_50_ < 0.1 mm) was used as a substrate, and a 5% Hoagland solution was provided as the nutrient media. In the experimental tanks, the air was steadily bubbled from the bottom to maintain sufficient carbon dioxide and oxygen in the water. Algae-free stocks were carefully selected for the experiments.

#### Setting up different light intensities

The light experiment was conducted by growing *P. anguillanus* (approximately 15 cm long) in four tanks (30 cm × 17.5 cm × 20.4 cm) (L × W × H) exposed to four different light intensities (50, 100, 200, and 300 µmol m^−2^ s^−1^ PAR intensity) from 6:00 a.m. to 6:00 p.m. These PAR intensities were chosen for the experiment to range in the field conditions. The control condition was maintained in another tank by keeping the plants in preincubation. Before starting the experiment, three individual plants were sampled from each tank. Then they were sampled every 5 days for 30 days at 1:00 p.m. (7 h after first light), and their stress assays were conducted.

#### Salinity test

In salinity experiments, *P. anguillanus* (approximately 15 cm long) was grown in four 15.7 cm × 15.7 cm × 24 cm (L × W × H) tanks, with 0, 5, 10, and 15‰ salinity (NaCl). A 5% Hoagland solution was added for the nutrient source. Water temperature was maintained at 25 ± 2 °C and PAR intensity of 100–120 μmol m^−2^ s^−1^ with a 12 h/12 h photoperiod (light: 6:00 a.m.–6:00 p.m.; and dark: 6:00 p.m.–6:00 a.m.). In this set of experiments, the samples were analyzed every five days for 30 days at 1 p.m. to observe the transition from the start of the experiment.

#### Diurnal solar radiation

In another experiment, the H_2_O_2_ concentration’s response to diurnal solar radiation was investigated. Two *P. anguillanus*-grown tanks (30 cm × 17.5 cm × 20.4 cm), filled with water (28 ± 3 °C) and added 5% Hoagland solution, were located outdoors, either exposed to solar radiation or completely shaded. Three plants were sampled from each tank before and after solar radiation exposure, together with PAR measurement of the exposure tank. The solar radiation was 450 μmol m^−2^ s^−1^, 460 μmol m^−2^ s^−1^, 600 μmol m^−2^ s^−1^, 800 μmol m^−2^ s^−1^, 430 μmol m^−2^ s^−1^ PAR, and zero at each sampling time, respectively.

Plant lengths were measured using a millimeter scale at 5-day intervals. The SGR was calculated as the difference in the shoot length between the two observations. The SGR was obtained by dividing the length by the duration and was expressed in cm/day. At the experiment’s end, the plants were oven-dried at 70 °C for 72 h. The dry weight (DW) of the shoots was measured to confirm the reliability of the shoot length as a reference parameter of the growth rate. The weight/length ratio was 4.0 ± 1.0 mgDW/cm, regardless of conditions, except for the dying samples. Thus, SGR values were used as the reference growth rate^68^.

### Chemical analyses

The Chl-*a*, Chl-*b*, and total CAR contents were spectrophotometrically (UV Mini 1210, Shimadzu, Japan) determined by extracting pigments of *N*, *N*-dimethylformamide after being kept in the dark for 24 h. The results were expressed in fresh weight (FW)^69,70^. The chlorophyll fluorescence parameters were measured by fluorescence imaging (FC 1000-H, Photon Systems Instruments, Czech Republic) with auto-image segmentation. Initially, the plants were dark-adapted for 20 min, and the maximum quantum efficiency of PSII (*F*_*v*_/*F*_*m*_) was obtained.

The stress assay compounds H_2_O_2_, CAT, APX, and POD were extracted by grinding the freeze-dried (with liquid nitrogen) fresh plant sample (about 50 mg) with an ice-cold, pH 6.0, 50 mM phosphate buffer. Polyvinylpyrrolidone (PVP) was added to the extraction to mask the effect of phenolic compounds in the plant materials. Then, the extractions were centrifuged at 5000×*g* and 4 °C for 15 min, and the supernatant was separated and incubated at − 80 °C for further analysis. In each treatment, the extractions were performed in triplicate. All the results were expressed in FW.

The H_2_O_2_ contents were determined calorimetrically following the TiSO_4_ method^71^, with modifications, as the most stable results were obtained compared with other methods. The reaction mixture contained 750 µL of enzyme extract and 2.5 mL of 1% TiSO_4_ in 20% H_2_SO_4_ (v/v), which was centrifuged at 5000×*g* and 20 °C for 15 min. The optical absorption of the developed yellow color was measured spectrophotometrically at a wavelength of 410 nm. The H_2_O_2_ concentrations in the samples were determined using the prepared standard curve for known concentration series. The H_2_O_2_ contents were expressed in µmol/g FW.

The absorption at 410 nm may include the effect of other soluble compounds^72,73^. Thus, the H_2_O_2_ concentration was calculated from the slope of the standard curve obtained from the known H_2_O_2_ concentration, which was offset, derived by the intercept absorption rate with zero H_2_O_2_ concentration samples^71^. The results were compared with those of the e-FOX method^73^, and a suitable correlation (*r* = 0.98) was obtained. The results were presented as μmol/g FW.

The CAT activity was measured as follows: 100 µL of 10 mM H_2_O_2_ and 2.0 mL of 100 mM potassium phosphate buffer (pH 7.0) were added to the cuvette before 500 µL of enzyme extract was added to initiate the reaction. The optical absorbance reduction at 240 nm was recorded every 10 s for 3 min. Finally, the CAT activity was obtained using an extinction coefficient^74^ of 40 mM^−1^ cm^−1^.

The APX activity was determined as follows: the reaction mixture contained 100 µL of enzyme extract, 200 µL of 0.5 mM ascorbic acid in 50 mM potassium phosphate buffer (pH 7.0), and 2 mL of 50 mM potassium phosphate buffer (pH 7.0). The reaction was initiated by adding 60 µL of 1 mM H_2_O_2_. The decrease in absorbance at 290 nm was recorded every 10 s. The APX activity was calculated using an extinction coefficient of^75^ 2.8 mM^−1^ cm^−1^. The POD activity was spectrophotometrically measured based on the oxidation of guaiacol with the presence of H_2_O_2_. The reaction mixture contained 3.0 mL of pH 6.5 potassium phosphate buffer, 40 µL of 30 mM H_2_O_2_, and 50 µL of 0.2 M guaiacol. The reaction was initiated by adding 100 µL of crude enzyme extract, and the increase in absorbance at 420 nm was recorded every 10 s for 3 min. The absorbance change rate and POD activity were calculated using an extinction coefficient^76^ of 26.6 mM^−1^ cm^−1^.

### Statistics

The data analyses were carried out by using the R statistical package^77^. Data were tested for normality with the Shapiro–Wilk method before statistical analyses. Kruskal–Wallis tests were performed to compare the performance variation of *P. anguillanus* under different solar light and salinity regimes. Spearman’s rank-order correlation method was used to evaluate the correlations among study parameters. Bonferroni post hoc was done to confirm the pairwise significance level.

### Ethical permission

This study has been approved by the Ministry of Land, Infrastructure, Transport, and Tourism of Japan (the River Works Technology Research and Development program) and by Shimane Prefecture (Hozen-Saisei Kyogikai of Lake Shinji).

## Supplementary Information

Supplementary Information.

## Data Availability

The authors highly appreciate and state that data is available for everyone in the supplementary file named Raw Data.
